# Entropy Generation Analysis of Laminar Flows of Water-Based Nanofluids in Horizontal Minitubes under Constant Heat Flux Conditions

**DOI:** 10.3390/e20040242

**Published:** 2018-04-02

**Authors:** Mehrdad Karimzadehkhouei, Mostafa Shojaeian, Abdolali Khalili Sadaghiani, Kürşat Şendur, M. Pinar Mengüç, Ali Koşar

**Affiliations:** 1 Mechatronics Engineering Program, Faculty of Engineering and Natural Sciences, Sabanci University, Tuzla, Istanbul 34956, Turkey; 2 Center of Excellence for Functional Surfaces and Interfaces for Nano Diagnostics (EFSUN), Sabanci University Nanotechnology and Applications Center (SUNUM), Sabanci University, Tuzla, Istanbul 34956, Turkey; 3 Department of Mechanical Engineering, Ozyegin University, Cekmekoy, Istanbul 34782, Turkey

**Keywords:** entropy generation, heat transfer coefficient, TiO_2_ and Al_2_O_3_ nanoparticles, carbon nanotubes, nanofluid, minitube

## Abstract

During the last decade, second law analysis via entropy generation has become important in terms of entropy generation minimization (EGM), thermal engineering system design, irreversibility, and energy saving. In this study, heat transfer and entropy generation characteristics of flows of multi-walled carbon nanotube-based nanofluids were investigated in horizontal minitubes with outer and inner diameters of ~1067 and ~889 µm, respectively. Carbon nanotubes (CNTs) with outer diameter of 10–20 nm and length of 1–2 µm were used for nanofluid preparation, and water was considered as the base fluid. The entropy generation based on the experimental data, a significant parameter in thermal design system, was examined for CNTs/water nanofluids. The change in the entropy generation was only seen at low mass fractions (0.25 wt.% and 0.5 wt.%). Moreover, to have more insight on the entropy generation of nanofluids based on the experimental data, a further analysis was performed on Al_2_O_3_ and TiO_2_ nanoparticles/water nanofluids from the experimental database of the previous study of the authors. The corresponding results disclosed a remarkable increase in the entropy generation rate when Al_2_O_3_ and TiO_2_ nanoparticles were added to the base fluid.

## 1. Introduction

Thermal management of microelectromechanical systems (MEMS) has been one of the most popular research areas during the last decade. Removing more heat from smaller spots with microchannel heat sinks is one of the important application areas in cooling of electronic devices. In classical methods, air, water, and liquid coolants such as refrigerants were utilized. There are many applications of liquid-based coolants in small scale such as electronics cooling [1], micro heat exchangers [2], renewable energy systems such as solar energy systems [3], and bioengineering applications. Significant parameters affecting the heat transfer performance are size, surface roughness, working fluid and surface geometry. Using conventional fluids such as water, engine oil and ethylene glycol as working fluids is not very desirable because of their rather average thermophysical properties. One of the methods for enhancing the thermophysical properties of the mentioned fluids is by adding metallic or non-metallic particles. Recently, nano-size solid particles called nanoparticles have been employed to increase the performance [4]. This preference is not only because of the unique properties afforded by their small size and high surface to volume ratio [5] and nanoparticles’ outstanding thermophysical properties, but also because of their better suspension ability compared to microparticles. Nanofluids prepared using nanosized metallic or non-metallic solid particles in conventional base fluids display significantly enhanced thermal conductivity and heat transfer capability compared to their pure base fluid [6,7]. An important parameter in nanofluids is the shape of nanoparticles in the base fluid [8].

There are a number of studies related to convective heat transfer characteristics in microchannels [9] and heat transfer of nanofluids in mini/microchannels with the use of different types of nanoparticles such as Al_2_O_3_ nanoparticles in water [10,11,12,13,14], TiO_2_ nanoparticles in water [14], CuO nanoparticles in R-113 refrigerant [15,16], CuO and SiO_2_ nanoparticles in R-134a refrigerant [17] and Cu nanoparticles in water. Recently, the stability of nanofluids with multi-wall carbon nanotubes (by adding surfactant) and their thermal conductivities were investigated [18,19]. Kumaresan et al. [20] studied heat transfer characteristics of CNT-based nanofluids in a tubular heat exchanger with inner and outer tubes diameters of 12.7 mm and 25.4 mm and various lengths, respectively, for cooling/heating applications. They claimed that Nusselt number for the nanofluids increased with a decrease in Reynolds number as the multi-walled carbon nanotube (MWCNT) concentration increased. Ding et al. [21] investigated heat transfer performance of CNT-based nanofluids flowing through a copper circular tube with an inner diameter of 4.5 mm and a length of 970 mm. Significant heat transfer enhancements were observed at a mass fraction of 0.5 wt.% and Reynolds number of 800. Meyer et al. [22] experimentally investigated convective heat transfer enhancement of aqueous suspensions of multi-walled carbon nanotubes flowing through a straight horizontal copper tube with an inner diameter of 5.16 mm for a Reynolds number range of 1000–8000, which also includes the transitional flow regime. According to their results, enhancement was more clearly observed in the turbulent regime.

In another minichannel configuration with an inner diameter of 6 mm [23], heat transfer coefficients were increased nearly by 50% by adding nanotubes with a concentration of 0.5 vol.% to deionized water under laminar flow conditions. An application-based study focused on CPU cooling performance by using alumina nanoparticles and carbon nanotubes [24]. The best CPU cooling performance improvement (by nearly 13%) was obtained at a concentration of 0.25 vol.% for carbon nanotubes. Elnajjar et al. [25] obtained the optimum fraction ratio of multi-walled CNTs inside a microchannel with a diameter of 800 µm as 0.1wt.% in developing laminar flows. Utomo et al. [26] investigated the convective heat transfer of Al_2_O_3_, TiO_2_ and CNT nanoparticle-water nanofluids. Their results indicated that adding nanoparticles to water only enhanced heat transfer up to 10%, which was still within the error bars of the correlations developed for pure liquids. Ashtiani et al. [27] changed the geometry of a circular tube with an inner diameter of 14.5 mm to flattened tube (oblong shape). They performed their heat transfer experiments on nanofluids with mass fractions up to 0.4 wt.%. According to their experimental results, circular tubes had better results, and higher mass fraction resulted in more enhancement.

Most of the studies related to entropy generation on nanofluid flows were numerical or theoretical and considered various nanoparticles and geometries, such as circular, rectangular or square mini- or micro-channels, flat plate solar collector, and co-rotating cylinders, while there are rather few experimental studies on this topic particularly for small channels/tubes. Nanoparticles extensively utilized in the recent studies include Al_2_O_3_ [28,29,30,31,32,33,34,35], CuO [30,36,37,38], and Cu [39] nanoparticles, which were dispersed in water or other base fluids, such as ethylene glycol (EG), i.e., Al_2_O_3_/EG and TiO_2_/EG [40], or refrigerants, i.e., Al_2_O_3_, CuO, SiO_2_, and MgO nanoparticles mixed in HFE7000 [41].

Mahian et al. [40] analytically analyzed entropy generation of nanofluids in co-rotating cylinders at constant heat flux on the wall. They utilized two nanoparticles, namely Al_2_O_3_ and TiO_2_ nanoparticles, dispersed in ethylene glycol with volume fractions up to 5% and compared the effect of nanoparticles type in second law analysis. According to their results, increasing the nanofluid volume fraction lead to a decrease in Bejan number and a decrease in entropy generation. The effects of porous media and related parameters, such as porous layer thickness and its positions, for Al_2_O_3_ nanoparticles/water nanofluid flows inside an annular pipe on both first and second law analysis were investigated by Siavashi et al. [29]. According to their entropy generation results, an increase in nanoparticles concentration, porous layer thickness, or flow rate reduced thermal entropy generation and raised frictional entropy generation. Besides, the effect of Al_2_O_3_ nanoparticles/water and CuO nanoparticles/water nanofluids on heat transfer and entropy generation in a rectangular microchannel heat sink with longitudinal vortex generators (LVGs) was numerically studied by Ebrahimi et al. [30]. Their first law results reported enhancements in heat transfer with the use of both nanofluids and with the decrease in the size of Al_2_O_3_ nanoparticles in the mentioned heat sink with LGVs. The maximum enhancement in heat transfer was observed for CuO nanoparticles/water nanofluid with the highest volume fraction, 3%, nanoparticle diameter of 29 nm. Additionally, according to the second law analysis, the authors proposed nanofluids as an excellent option for heat transfer applications and enhancing the performance.

The review of Mahian et al. [42] covered studies related to the entropy generation for nanofluid flows in different geometries, such as microchannels, square, circular, and coiled conduits etc., and also included models for calculations of nanofluids thermophysical properties. According to this study, the study of entropy generation estimation for nanofluids based on experimental studies would provide a good contribution to the literature. To the authors’ best knowledge, Singh et al. [43] study is one of few scarce entropy generation studies based on experimental data for Al_2_O_3_ nanoparticles with the diameter of 45 nm dispersed in water and with volume fractions of 0.25 vol.%, 0.5 vol.%, and 1 vol.% in two microchannels with hydraulic diameters of 218 µm and 303 µm. According to their results, the thermal part of entropy generation played the major role. For the smaller channel diameter, an increase in the concentration caused an increase in entropy generation, whereas opposite trends existed for the bigger channel diameter. Additionally, theoretical entropy generation was lower than the experimental one possibly due to the dominance of frictional entropy, and the total entropy generation of bigger channel diameter was more due to dominance of the thermal part of entropy generation.

There are a few studies in the literature investigating convective heat transfer in thermally developing flows of multi-walled carbon nanotube (MWCNT) based nanofluids in tubes with hydraulic diameters smaller than 1 mm. To provide more insight, in this study, forced convective heat transfer with MWCNTs, having an outer diameter of 10–20 nm and a length of 1–2 µm, which were added to pure water with mass fractions of 0.25%, 0.5% and 1%, was investigated in a minitube with inner and outer diameters of ~889 and ~1067 µm, respectively. 

To improve the efficiency of a thermal system, entropy generation minimization is essential, as a system with less entropy generation (due to irreversibilities in fluid friction or heat transfer) is desirable. For nanofluids as a candidate for heat transfer enhancement, the entropy generation analysis becomes vital. Therefore, because the entropy generation analysis based on the experimental results was not extensively covered in the literature, second law analysis with entropy generation of CNTs/water nanofluids of this study as well as of Al_2_O_3_ and TiO_2_ nanoparticles/water nanofluids of the previous study [14] of the authors was performed.

## 2. Nanofluid Preparation, Characterization and Thermophysical Properties

The most widely used and economical method for nanofluid preparation is the two-step method [44]. In this method, first, dry powder is produced using physical and chemical reactions. In the second step, prepared nanoparticles are mixed in the base fluid. Aggregation tendency of nanoparticles is considered as one of the disadvantages of this method [44].

**Figure 1:**
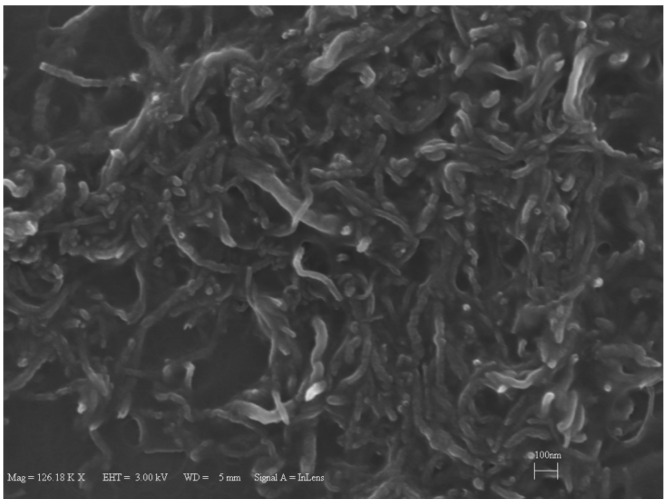
SEM image of carbon nanotubes (CNTs)/water nanofluid sample.

In this study, the nanofluids were prepared using the two-step method. To prevent aggregation 2500 ppm sodium dodecyl sulfate (SDS) was added to the base fluid as surfactant. The outer diameter of the multi-wall carbon nanotubes (Ionic Liquids Technologies, IoLiTec GmbH, Heilbronn, Germany) is 10–20 nm, and their length is 1–2 µm. Stirring and sonication were performed for 1 h in a sonication bath. A sample scanning electron microscopy image (SEM) of CNTs is shown in Figure 1, which was obtained as follows: After evaporation of droplets of the CNTs-based nanofluids (using an oven), the residue was subjected to scanning electron microscopy (SEM) examination.

**Figure 2:**
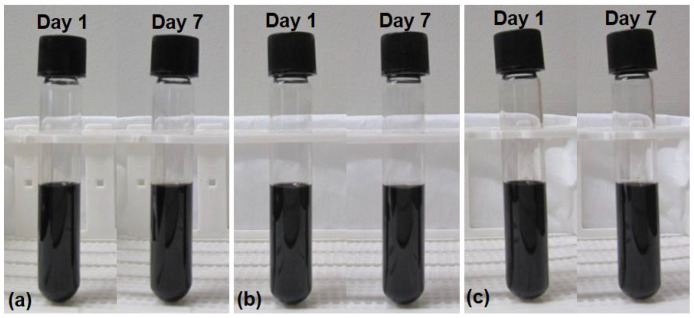
Prepared CNTs/water nanofluids during one-week period for mass fraction ratios of: (**a**) 0.25 wt.%, (**b**) 0.5 wt.%, and (**c**) 1 wt.%.

Prior to the experiments, images of the prepared samples, which were filled in collection tubes, were taken for a period of one week to investigate the stability of the nanofluids, as shown in Figure 2. No sedimentation was observed for any of the samples (0.25, 0.5 and 1 wt.%), implying the stability of the prepared nanofluids. Therefore, nanofluids were assumed to be stable for a short period of time (at least between the nanofluid preparation and experiments).

Thermophysical properties of the nanofluids were calculated using widely used expressions in the literature. Nanofluid density [45] is found as:

(1) $$\rho_{nf}=\left(1-\varphi \right)\rho_{bf}+\varphi \rho_{np},$$

where *ϕ* is the volume fraction and *ρ_nf_*, *ρ_bf_* and *ρ_np_* are the nanofluid, base fluid and nanoparticle densities, respectively (the density of CNT is *ρ_np_* = 2.1 g·cm^3^ [46]).

Nanofluid viscosity [47] is calculated as:

(2) $$\mu_{nf}=\mu_{bf}\left(1+2.5\varphi \right),$$

where *μ_nf_* and *μ_bf_* are the nanofluid and base fluid viscosities, respectively. 

Nanofluid specific heat capacity [48] is obtained as:

(3) $$C_{p,nf}=\left(1-\varphi \right)C_{p,bf}+\varphi .C_{p,np},$$

where *C_p,nf_*, *C_bf_* and *C_p,np_* are the specific heat capacities of nanofluid, base fluid and nanoparticles, respectively (the specific heat capacity of CNT is taken as *C_p,np_* = 0.6 kJ/kg·K [49]).

## 3. Experimental Setup and Procedure

**Figure 3:**
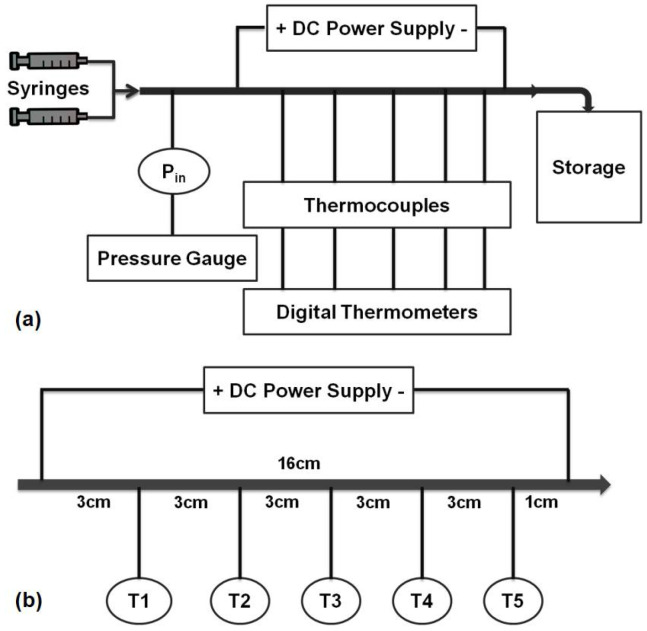
Schematic of the: (**a**) experimental setup, and (**b**) test section.

The experimental setup, shown in Figure 3a, consists of a syringe pump (Cole-Parmer 200 Touch Screen Series, Vernon Hills, IL, USA) for the delivery of nanofluids to the test section, a pressure gauge (Omega Engineering Inc., Stamford, CT, USA) for measuring the inlet pressure of the test section, a DC power supply (Xantrex Technology Inc., Elkhart, IN, USA) for applying the desired heat flux with the integration of two alligator clips on the minitube (joule heating of 16 cm heated test section), 5 T-type thermocouples (Omega Engineering Inc.) at specified locations (as displayed in Figure 3b) for the surface temperature measurements at different locations (inlet, middle and outlet) of test section, and a hypodermic stainless steel tube (Small Parts Inc., Logansport, LA, USA) as the test section with inner and outer diameters of 889 µm and 1067 µm, respectively. Since only one set of experiments was performed on each mini-tube, the experimental setup was designed as an open loop system, and the working fluid was collected to a reservoir at the end of the test section. The inlet flow rate was controlled via the controller of the syringe pump. In order to be sure that the pressures and local temperatures were acquired under steady state conditions, the data acquisition was initiated after a sufficiently long period of time.

The typical experimental procedure has the following steps: (a) preparing the nanofluids via stirring and sonication, (b) characterization of the nanofluids by the Scanning Electron Microscopy technique, (c) pumping the nanofluid through the microtube with a syringe pump, (d) heating the microtube by using two alligator clips at the desired heat flux, (e) measuring the inlet pressure by using the pressure transducer and calculating friction factors, (f) measuring the temperatures of the outer surface of the microtube using thermocouples, which were attached on the surface and sealed with thermal paste, (g) calculating the temperature of the microtube inner surface and fluid temperature based on the fluid inlet temperature and measured outer surface, respectively, and finally, (h) calculating the heat transfer coefficients based the general convective heat transfer coefficient equation.

## 4. Data Reduction 

Measured data from the pressure gauge, thermocouples and DC power supply were used to calculate the friction factor, inner wall and fluid temperatures, heat transfer coefficient, and entropy generation rate.

The experimental friction factor, *f*, is calculated as:

(4) $$f=\frac{2\Delta PD_{i}}{L_{tot}\rho_{nf}u^{2}},$$

where Δ*P* is the pressure drop, *D_i_* is the inlet diameter, *L_tot_* is the minitube total length and u is the streamwise flow velocity.

The local inner surface temperature, *T_w,j_*, is expressed as:

(5) $$T_{w,i}=T_{w,o}+\frac{{\dot{q}}_{net}}{4k_{ss}}\left(\frac{D_{o}^{2}}{4}-\frac{D_{i}^{2}}{4}\right)-\frac{{\dot{q}}_{net}}{2k_{ss}}\frac{D_{o}^{2}}{4}\ln\left(\frac{D_{o}}{D_{i}}\right),$$

where *T_w.o_* is the outlet wall temperature, ${\dot{q}}_{net}$ is the volumetric net heat flux, *k_ss_* is the stainless steel thermal conductivity, *D_o._* and *D_i_* are the outer and inner diameters, respectively.

The volumetric heat generation rate is obtained as:

(6) $${\dot{q}}_{net}=\frac{P_{el}-Q_{loss}}{\pi \left(\frac{D_{o}^{2}}{4}-\frac{D_{i}^{2}}{4}\right)L_{h}},$$

where *P_el_* is the electrical power, *Q_loss_* is the heat loss and L*_h_* is the heated length. 

The local fluid temperature, *T_f,i_*, is found using the energy balance for the fluid as:

(7) $$T_{f,i}=T_{in}+\frac{\left(P_{el}-Q_{loss}\right)x_{th}}{\dot{m}C_{p,nf}L_{h}},$$

The local heat transfer coefficient, *h*, is deduced as:

(8) $$h=\frac{P_{el}-Q_{loss}}{A_{s}\left(T_{w,i}-T_{f,i}\right)},$$

where *T_w,i_* is the inner wall temperature, *T_f,i_* is the fluid temperature and *A_s_* is the minitube heated surface area which is calculated as:

(9) $$A_{s}=\pi \cdot D_{i}\cdot L_{h},$$

The uncertainties in experimental parameters were provided by the manufacturer’s specification sheets and/or obtained using the propagation of uncertainty method included in the study of Kline and McClintock [50] as:

(10) $$U_{R}={\left[{\left(\frac{\partial R}{\partial x_{1}}U_{1}\right)}^{2}+{\left(\frac{\partial R}{\partial x_{2}}U_{2}\right)}^{2}+\ldots +{\left(\frac{\partial R}{\partial x_{n}}U_{n}\right)}^{2}\right]}^{1/2},$$

where $R=R(x_{1},x_{2},\ldots ,x_{n})$ is a function of experimental parameters. The obtained uncertainties in electrical power, flow rate, friction factor and heat transfer coefficients are ±0.32%, ±0.4%, ±1.4% and ±3.2%, respectively. 

For internal flows, Bejan [51] defined the equation for the rate of entropy generation per unit length as follows:

(11) S˙′gen=q″2πD2kT2Nu((Re)D,Pr)+8m˙3πD2π2ρ2Tf((Re)D)D5=(S˙′gen)heat−transfer+(S˙′gen)fluid−friction

where *Nu* is the Nusselt number, *Nu* = *hD_i_*/*k_f_*, and $\dot{m}$ is the mass flow rate. Therefore, in this study, the entropy generation rate is calculated based on Equation (11), which is a function of Nusselt number and friction factor obtained from the experimental data. 

The Bejan number, Be, defined as the ratio of the entropy generated due to the heat transfer to the total entropy generation rate, is calculated as:

(12) $$Be=\frac{{\left({{\dot{S}}^{\prime }}_{gen}\right)}_{heat-transfer}}{{\left({{\dot{S}}^{\prime }}_{gen}\right)}_{heat-transfer}+{\left({{\dot{S}}^{\prime }}_{gen}\right)}_{fluid-friction}},$$

## 5. Results and Discussions

### 5.1. Convective Heat Transfer Analysis

**Figure 4:**
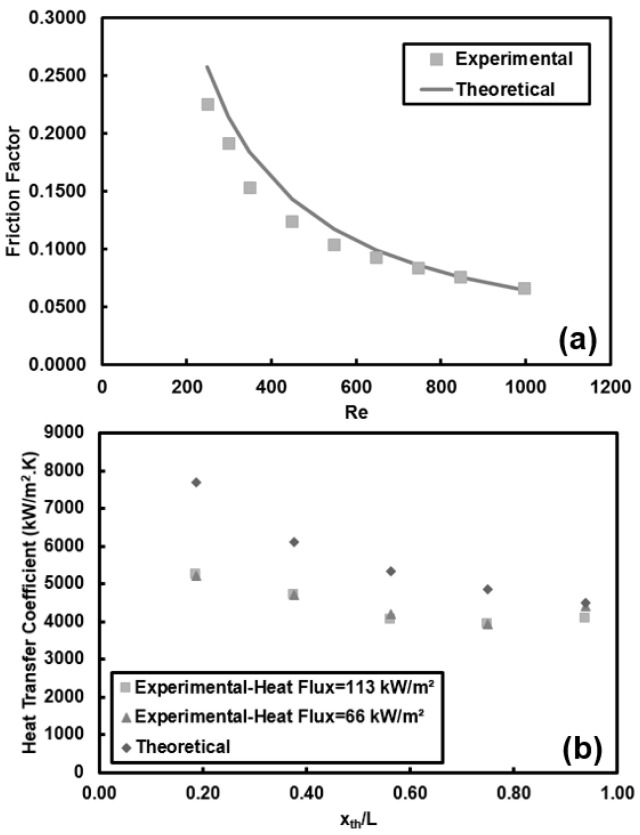
Validation of the experimental setup with pure water for: (**a**) friction factor, and (**b**) heat transfer coefficient at different applied heat fluxes and Reynolds number of 1000.

The experimental setup and data reduction process are validated by comparing the experimental heat transfer coefficients and friction factors with available correlations in the literature. For example, for the case of *Re* = 1000 at the heat flux of 66 kW/m^2^ the comparison is demonstrated in Figure 4. 

All the pure water data can be predicted within ±15% by the theoretical friction factor correlation recommended for fully developed laminar flows in circular tubes (*f* = 64/*Re*, Figure 4a). Furthermore, obtained heat transfer coefficients, Figure 4b, for pure water are compared to the Shah and London [52] correlation, which is applicable for thermally developing and hydrodynamically fully developed flows. In this comparison, the pure working fluid case (de-ionized water) was considered, and the heating length was kept as 16 cm. The data can be predicted within ±30% by this correlation. It should be noted that the theoretical heat transfer coefficient is calculated according to the local thermophysical properties of fluid in the tube. One of the major reasons for deviations is that the experiments were performed in tubes with much smaller diameter than the conventional tubes. Additionally, as seen in Figure 4 the difference between experimental results and those predicted by correlations decreases along the heated length. One major reason for deviations in the entrance region is related to rather higher uncertainties in cases with small heated length and small applied power.

**Figure 5:**
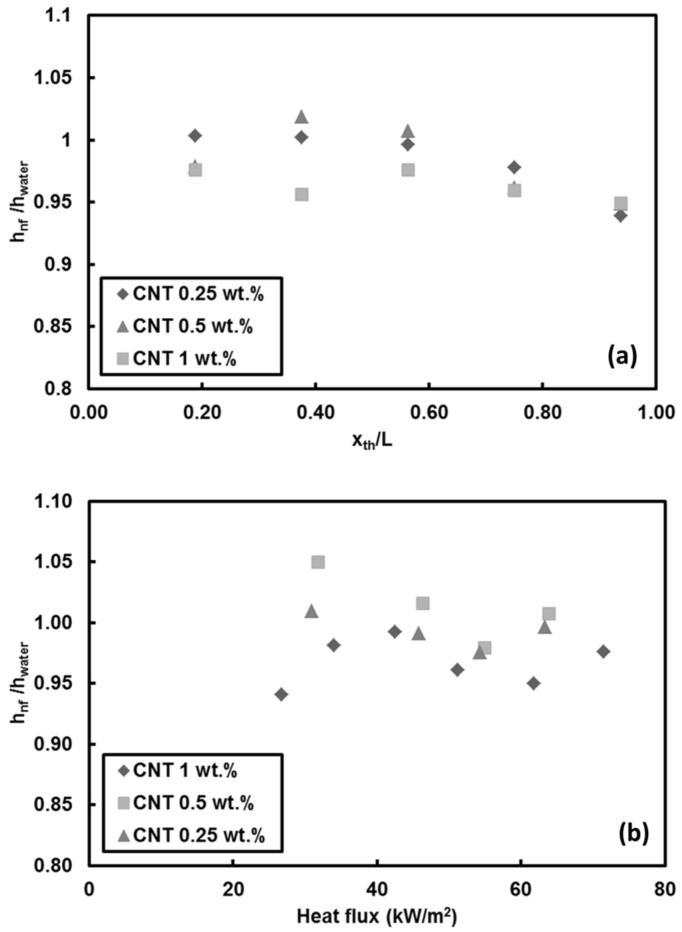
Local heat transfer coefficients of CNTs/water nanofluids for: (**a**) all the thermocouples (q″ = 66 kW/m^2^), and (**b**) the third thermocouple (*Re* = 500).

The experiments were divided into two sets of Reynolds numbers, namely 500 and 1000 and three mass fractions sets of 0.25, 0.5 and 1 wt.%. Local heat transfer coefficients of CNTs nanofluids are shown in Figure 5. 

At low Reynolds number (*Re* = 500), except for a slight degradation in the heat transfer coefficient for CNTs/water nanofluid at the mass fraction ratio of 1 wt.%, the ratio of heat transfer coefficient for the nanofluid case to heat transfer coefficient for the pure water case is close to the unity. This means that the heat transfer coefficient remains almost the same with the introduction of CNTs to the base fluid for all the chosen fractions. Although adding CNTs to the base fluid decreases the thermal boundary layer thickness (by enhancing the thermal conductivity of the working fluid), obtained results revealed that at low Reynolds number, mass fraction has no considerable effect on heat transfer (Figure 5a). Soret effect and corresponding mass diffusion play a major role on particle distribution and heat transfer in thermally developing flows particularly near the inlet of the tube (due to higher temperature gradient) [53]. Furthermore, it was observed that initially the heat transfer ratio increases with x_th_/L, reaches a maximum at a location near the middle of the tube, and then decreases along the tube. Small hydraulic diameter and small heating length intensify the Soret effect, resulting in non-uniform nanoparticle dispersion in the working fluid. This is one of the reasons for a low heat transfer coefficient ratio (nanofluid/water).

**Figure 6:**
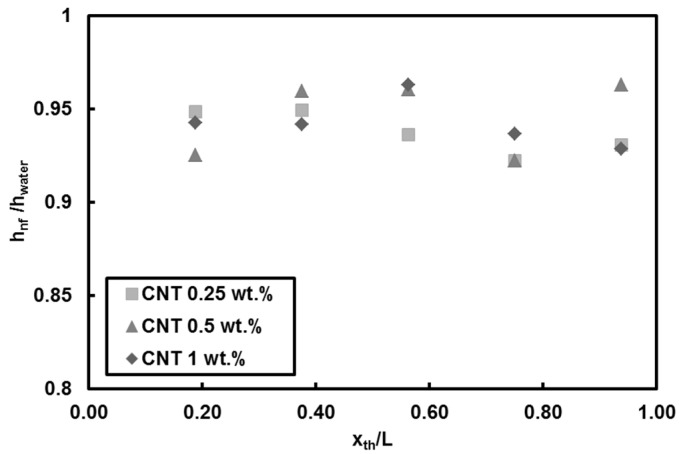
Local heat transfer coefficients of CNTs/water nanofluids (*Re* = 1000, q″ = 66 kW/m^2^).

The temperature variations of third thermocouple (middle of the mini-tube) for nanofluids with different mass fractions are shown in Figure 5b. The heat transfer coefficient ratios converge to unity as the heat flux increases, implying that the CNTs fraction has no promising effect on heat transfer coefficient at the Reynolds number of 500. Local heat transfer coefficients for higher Reynolds number (*Re* = 1000) at heat flux of 66 kW/m^2^ are displayed in Figure 6. Unlike the low Reynolds number (*Re* = 500) results, there is a slight reduction in heat transfer, which is less than 8%.

**Figure 7:**
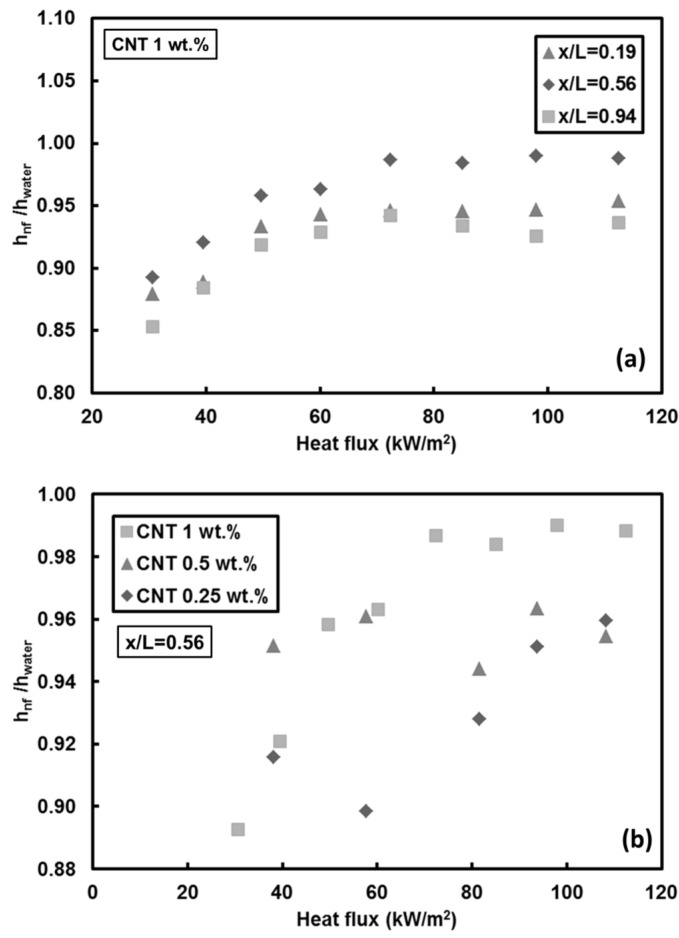
Local heat transfer coefficients for: (**a**) different thermocouple positions, and (**b**) at the middle of the test section (the third thermocouple (x/L = 0.56) (*Re* = 1000).

Figure 7 shows the obtained local (three locations of x/L = 0.19, 0.56 and 0.96 along the minitube) heat transfer coefficient for the nanofluid of with the mass fraction of 1 wt.%. At *Re* = 1000, as the heat flux increases, the heat transfer coefficient ratio (h_nf_/h_water_) increases, Figure 7a. This is associated with decreased viscosity of the fluid, which leads to better mixing of nanoparticles inside the base fluid. Furthermore, it can be observed that almost for all the experiments, the obtained heat transfer coefficients at the middle of the mini-tube (non-dimensional location of x/L = 0.56) are higher than those at other locations, Figure 7b. 

Heat transfer experiments are performed to investigate the effect of CNTs deposition due to the fact that the potential deposition of CNTs on the minitube surface is one of the parameters affecting the heat transfer performance. In these sets of experiments, firstly pure water was pumped into the bare minitube before performing the experiments with CNTs. Then, CNTs/water nanofluid experiments were performed to investigate the CNT based nanofluid convective heat transfer. Finally, the second set of experiments with pure water was performed on the tubes in which the nanofluid convective heat transfer were performed.

**Figure 8:**
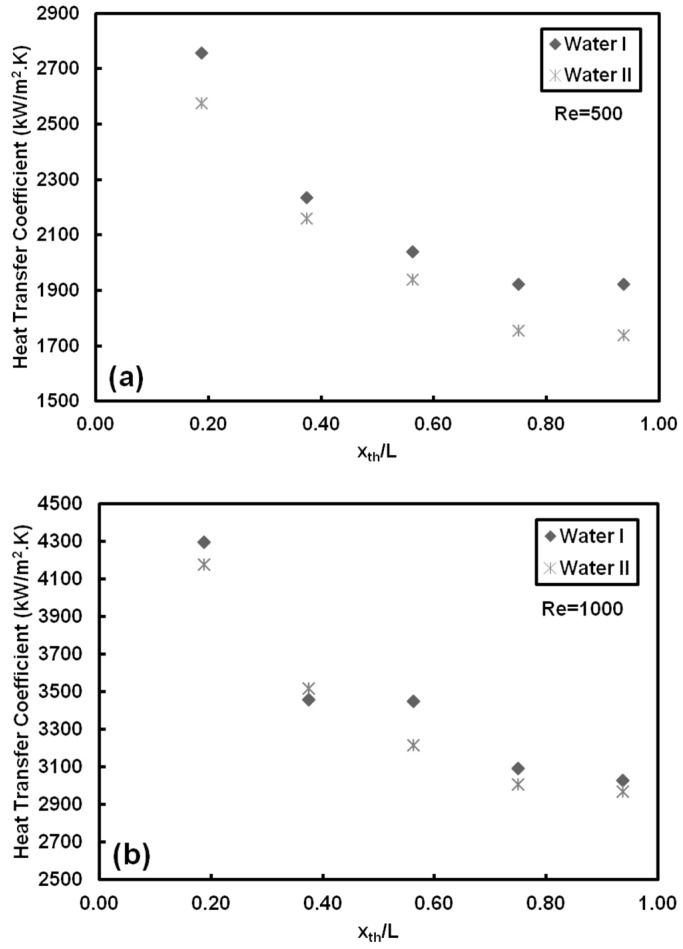
Local heat transfer coefficients of pure water before the nanofluid experiment (Water I), and after the nanofluid experiment in the same minitube (Water II) with coating via heating for: (**a**) *Re* = 500, and (**b**) *Re* = 1000.

Deposition on the inner surface of the minitube with CNTs slightly affects the heat transfer coefficient for both Reynolds numbers, Figure 8. It was found that the heat transfer coefficients of pure water decrease after CNT deposition on the inner surface the minitube. This is also one of the reasons for the reported heat transfer coefficient ratios close to unity (Figure 5, Figure 6 and Figure 7).

### 5.2. Second Law Analysis

It is well known that the total entropy generation contributes to two types of irreversibilities: Heat Transfer Irreversibility (HTI) and Fluid Friction Irreversibility (FFI). For these reasons, the improvement in thermal system performance in design and operation, a thorough investigation of second law analysis is necessary. Minimization of the entropy generation as a concept relying on the second law analysis serves for increasing the efficiency of thermal systems [54]. In this section, the second law analysis is considered, which is presented in terms of experimental data of entropy generation rate and Bejan number. For an efficient thermal system, these parameters resulting in minimum entropy generation are desirable. Bejan number, *Be*, reveals the contribution of each irreversibility in the analysis so that the convergence of Be to unity implies the domination of the heat transfer irreversibility, while its convergence to 0 implies the dominant contribution of the fluid friction.

#### 5.2.1. Entropy Generation of CNTs/Water Nanofluids Flows

**Figure 9:**
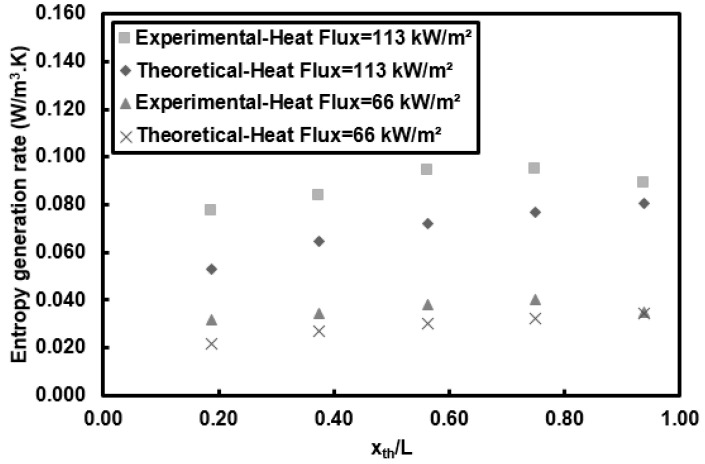
Comparison between experimental and theoretical entropy generation rates per unit length at different applied heat fluxes and Reynolds number of 1000.

A comparison of the experimental entropy generation rate per unit length of CNTs/water nanofluids and the case of *Re* = 1000 at the heat flux of 66 kW/m^2^ with the theoretical predictions, where the theoretical Nusselt number is calculated based on the Shah and London [52] correlation and the theoretical friction factor is Darcy friction factor (i.e., *f* = 64/*Re*), can be seen in Figure 9. The deviations of the experimental entropy generation rates from theoretical ones are lower than 50%. There is a good agreement between the experimental data and theoretical predictions for lower heat flux. A larger difference between experimental data and theoretical predictions is due to the fact that the theoretical entropy formulation is not purely theoretical due to presence of experimentally measured temperature, which changes the theoretical entropy values, as the theoretical temperatures are not available. 

The second law analysis is useful in terms of minimum entropy and maximum entropy principles. Minimum entropy analysis becomes significant in designing and improving the performance of thermal systems. Minimizing entropy generation helps to improve the energy efficiency of a system [54]. Maximum entropy principle has been applied in the field of spray and atomization, which has been successful in prediction of droplet size and velocity distributions. This approach for modeling of spray and atomization can be found in the pioneering studies of Sellens and Brzustowski [55] and Li and Tankin [56].

**Figure 10:**
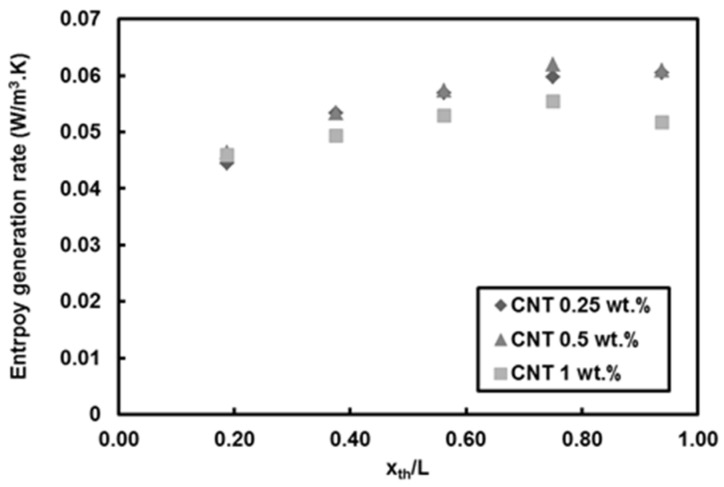
Local entropy generation rate of CNTs/water nanofluids (*Re* = 500, q″ = 66 kW/m^2^).

**Figure 11:**
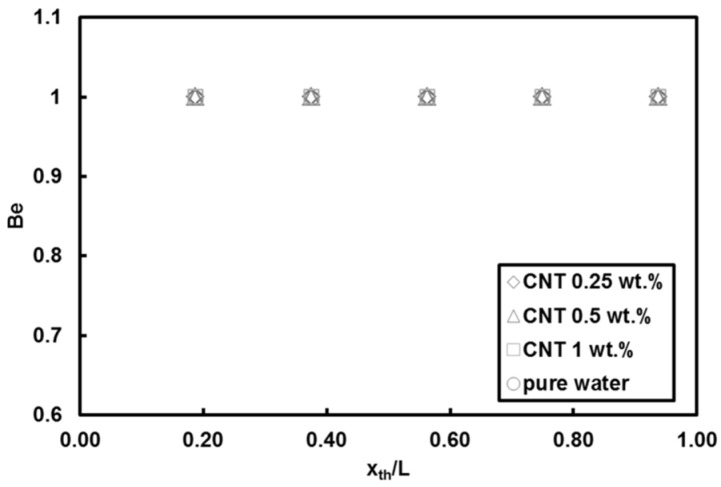
Local Bejan number of CNTs/water nanofluids (*Re* = 500, q″ = 66 kW/m^2^).

**Figure 12:**
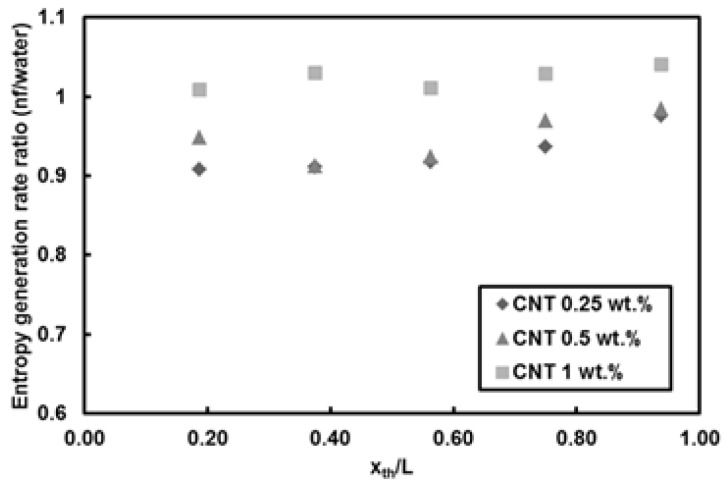
Local entropy generation rate of CNTs/water nanofluids relative to that of water (*Re* = 500, q″ = 66 kW/m^2^).

Figure 10 depicts the local entropy generation rate per unit length for different nanoparticle mass fractions at *Re* = 500 and heat flux of 66 kW/m^2^. The entropy generation rate monotonously increases with streamwise location, which is merely due to the decreasing trend in local Nusselt number along the minitube rather than the friction effect. The lower nanoparticle mass fractions (i.e., 0.25 wt.% and 0.5 wt.%) result more or less in the same entropy generation rate values, while the nanoparticle mass fraction of 1 wt.% leads to the lowest entropy generation rate, which is ascribed to the effect of enhanced thermal properties because of addition of nanoparticles to the base fluid. The reason for rather minor friction factor effect is explained in Figure 11, which shows the local Bejan number for different nanoparticle mass fractions at *Re* = 500 and heat flux of 66 kW/m^2^. It can be understood that the irreversibility is only due to the heat transfer as the Bejan number is unity for water and all nanoparticle mass fractions, thereby implying that friction factor has no role in the entropy generation rate for the water and nanofluids flows in the minitube under experimental conditions in this study. Figure 12 presents the comparison of entropy generation rate of nanofluid to that of pure water for *Re* = 500 and heat flux of 66 kW/m^2^. The entropy generation rate does not change by adding 1 wt.% CNTs with respect to water, even though a slight decrease in the heat transfer coefficient is seen for this case. In contrast, the nanoparticle mass fractions of 0.25 and 0.5 wt.% (i.e., addition of low amount of CNTs in pure water) cause a decrease in the entropy generation rate by up to 10%. Since the corresponding heat transfer coefficients do not vary at low nanoparticle mass fractions, the decrease could be only due to the enhanced thermophysical properties.

**Figure 13:**
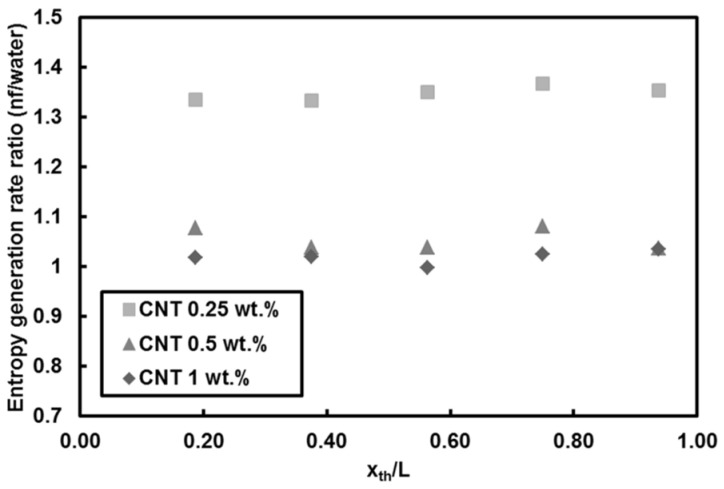
Local entropy generation rate of CNTs/water nanofluids (*Re* = 1000, q″ = 66 kW/m^2^).

The local entropy generation rate per unit length for different nanoparticle mass fractions at *Re* = 1000 and heat flux of 66 kW/m^2^ is exhibited in Figure 13. 

A different trend is observed for this case. For nanoparticle mass fractions of 1 and 0.5 wt.% fractions, there is no change in the entropy generation trend with respect to that of water. However, for 0.25 wt.%, the entropy generation rate remarkably increases (for about 35%). This behavior could be explained by the interplay between the heat transfer and thermal properties. For example, for the 0.25 wt.% case, the change in thermal property due to added CNTs cannot balance the corresponding decrease in heat transfer, and therefore, the entropy generation with CNTs increases relative to the pure water case.

**Figure 14:**
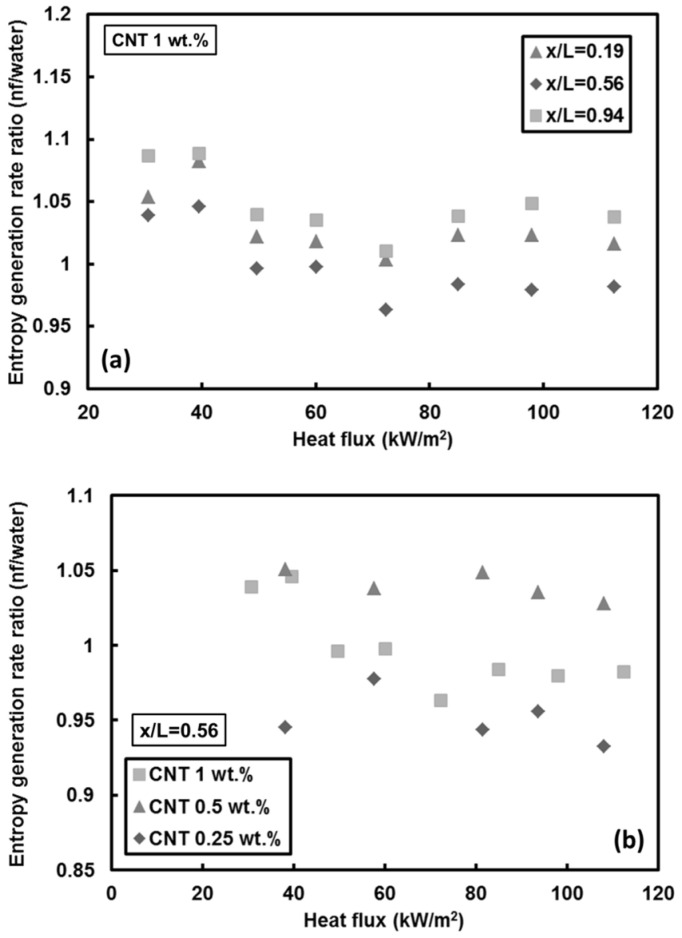
Local entropy generation rate for: (**a**) different thermocouple positions, and (**b**) the third thermocouple (x/L = 0.56) (*Re* = 1000).

The effect of heat flux on the entropy generation rate in the case of *Re* = 1000 for mass fractions 1 wt.% at different locations and for that for x_th_/L = 0.56 at different nanoparticle mass fractions are given in Figure 14a,b, respectively. It can be deduced that even though heat transfer increases to some extent with heat flux (for *Re* = 1000 case), the entropy generation rate is mainly independent of the heat flux for the range of heat flux under the experimental conditions studied here. This could be because of the reason that the increase in the heat flux predominates over the increased Nusselt number, according to the Equation (11).

#### 5.2.2. Entropy Generations in TiO_2_ and Al_2_O_3_ Nanoparticle/Water Nanofluids

**Figure 15:**
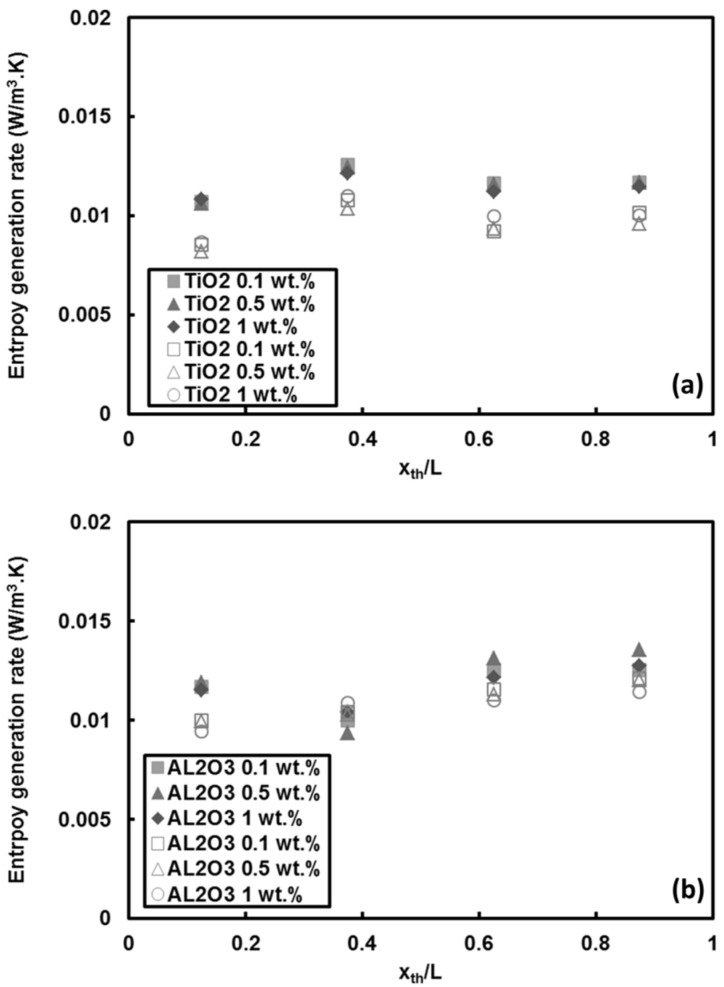
Local entropy generation rate of: (**a**) TiO_2_ nanoparticle/water, and (**b**) Al_2_O_3_ nanoparticle/water nanofluids at heat flux of q″ = 66 kW/m^2^ (filled symbols for *Re* = 530, open symbols for *Re* = 1070).

The entropy generation for TiO_2_ and Al_2_O_3_ nanoparticle/water nanofluid flows is presented in Figure 15. The heat transfer and pressure drop data were extracted from our previous work [14], in which the effect of these nanoparticles on heat transfer was examined for a minitube having inner and outer diameters of 502 and 717 μm. The local entropy generation rate at different mass fractions of TiO_2_ nanoparticles and Al_2_O_3_ nanoparticles for *Re* = 530 and 1070 are presented in Figure 15a,b, respectively. In contrast to CNTs, the entropy generation rate remains more or less constant with location for all mass fractions for both *Re*, which is due to the fact that the heat transfer reduction along the channel is balanced with the rise in the bulk temperature of the fluid. Also, the increase in the nanoparticle mass fractions does not have any considerable effect on the entropy generation rate. The increase in the Reynolds number, on the other hand, decreases the entropy generation, which is more visible for TiO_2_ nanoparticles. This reduction is because of the enhanced convective heat transfer at higher *Re* and is around 17% for TiO_2_ nanoparticles when the Reynolds number is raised from 530 to 1070.

**Figure 16:**
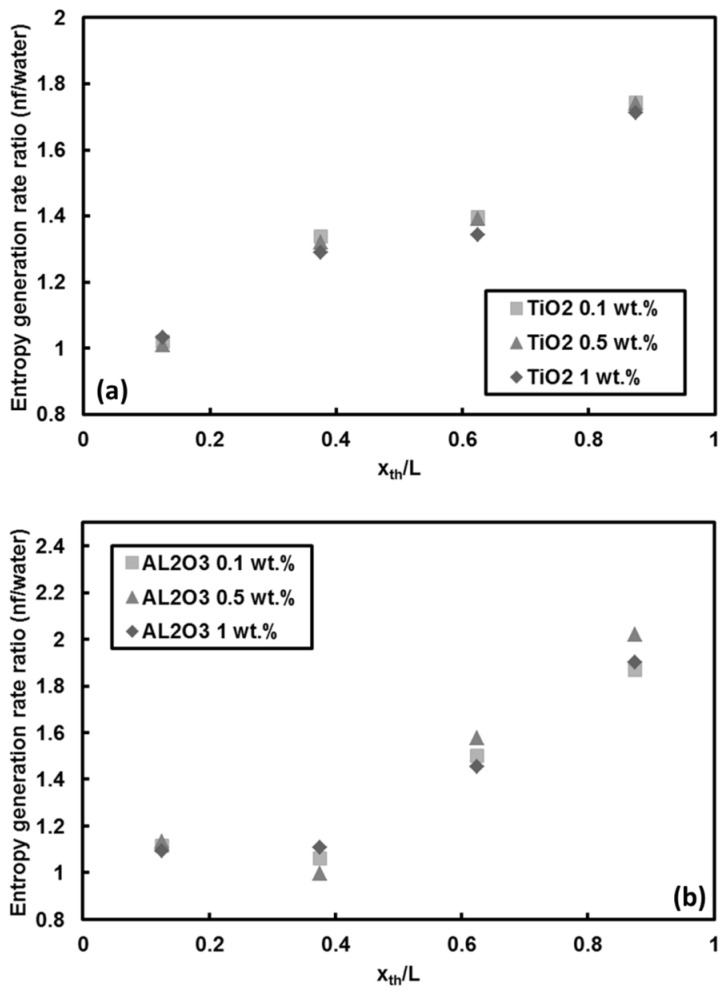
Local entropy generation rate ratio of water based: (**a**) TiO_2_, and (**b**) Al_2_O_3_ nanofluids versus water at *Re* = 1000 and q″ = 66 kW/m^2^.

The local entropy generation rate of TiO_2_ and Al_2_O_3_ nanoparticles/water nanofluids relative to pure water is displayed in Figure 16a,b, respectively, at *Re =* 530 and heat flux of 66 kW/m^2^. In contrast to the trends observed in the CNT case, it is clear that the entropy generation rate dramatically increases (by up to 100%) with addition of TiO_2_ and Al_2_O_3_ nanoparticles to the base fluid (e.g., water). Nevertheless, the increase in mass fraction itself has no effect on the entropy generation as mentioned earlier. The results also show that the effect of adding TiO_2_ and Al_2_O_3_ nanoparticles into water becomes more pronounced at more downstream locations, where the effect of convective heat transfer becomes much more significant. Furthermore, the calculated local Bejan numbers corresponding to the TiO_2_ and Al_2_O_3_ nanoparticles/water nanofluids were unity, implying the sole contribution of heat transfer irreversibility to the entropy generation (similar to Figure 11).

## 6. Conclusions

Convective heat transfer and entropy generation of nanofluid flows were investigated under laminar and thermally developing-hydrodynamically fully developed flow conditions at constant wall heat flux. Multi-wall carbon nanotubes (CNTs)/water nanofluids as well as two more traditional TiO_2_ and Al_2_O_3_ nanoparticles/water nanofluids were considered. 

Nanoparticles as heat transfer enhancement agents introduce a change in entropy generation (mainly excessive entropy generation) relative to pure water (base fluid). As thermal systems with minimized entropy generation are desirable in terms of energy efficiency improvement, entropy generation was experimentally investigated to offer more insight about how much change in entropy generation was generated as a result of nanoparticle addition into the base fluid. Major conclusions of this study are as follows:

- Adding CNT to pure water has no considerable effect on heat transfer coefficient for low Reynolds number (*Re* = 500). However, degradation in heat transfer was observed (by less than 10%) for high Reynolds number (*Re* = 1000). Deposition of CNT on the surface toward the end of the test section is the reason behind the decrease in heat transfer coefficient and slight deterioration in heat transfer near the exit. Soret effect and corresponding mass diffusion at the thermally developing flows play a major role on particle distribution and heat transfer due to higher temperature gradient especially near the inlet of the tube.
- Adding CNTs to the base fluid increases the thermal conductivity of the nanofluid. However, the results showed that mass fraction has no considerable effect on heat transfer at low Reynolds number because of short heating length. Small hydraulic diameter and small heating length intensify the Soret effect, resulting in non-uniform nanoparticle dispersion in the working fluid. This could be a possible reason for the low heat transfer coefficient ratio between the nanofluid and pure water.
- For CNTs/water nanofluids, the entropy generation rate remains almost unchanged at mass fraction of 1 wt.% (high nanofluid mass fraction). A reduction in the entropy generation rate (down to 10%) compared to pure water was observed at *Re* = 500 for mass fractions of 0.25 wt.% and 0.5 wt.% at *Re* = 1000. The only considerable increase in the entropy generation rate (about 35%) relative to pure water was seen for the lowest mass fraction of CNTs. In addition, heat flux did not exhibit any remarkable change in the entropy generation rate.
- For the other traditional nanofluids, the entropy generation rate pertinent to TiO_2_ and Al_2_O_3_ nanoparticles cases demonstrated that the entropy generation rate raised up to 100% at downstream locations. However, the increase in mass fraction of these nanoparticles (i.e., TiO_2_ and Al_2_O_3_) did not significantly change the entropy generation rate.
